# Asymptomatic and Mild SARS-CoV-2 Infections in a Hungarian Outpatient Cohort in the First Year of the COVID-19 Pandemic

**DOI:** 10.3390/tropicalmed8040204

**Published:** 2023-03-29

**Authors:** István Jankovics, Cecília Müller, Éva Gönczöl, Ildikó Visontai, István Varga, Márta Lőrincz, Dávid Kuti, Ágnes Hasitz, Péter Malik, Krisztina Ursu, Borbála Bányász, Júlia Sarkadi, Béla Dénes

**Affiliations:** 1 Department of Microbiology and Infectious Diseases, University of Veterinary Medicine Budapest, 1143 Budapest, Hungary; 2 Department of Chief Medical Officer, National Public Health Centre, 1097 Budapest, Hungary; 3 Division of Project Coordination, National Public Health Centre, 1097 Budapest, Hungary; 4 Division of Virology, Department of Reference Laboratory for Microbiology, National Public Health Center, 1097 Budapest, Hungary; 5 Family Doctor’s Office, 2000 Szentendre, Hungary; 6 Veterinary Diagnostic Directorate, National Food Chain Safety Office, 1143 Budapest, Hungary

**Keywords:** SARS-CoV-2, COVID-19, outpatient cohort, seroprevalence, mild infections, asymptomatic cases, first year of the pandemic, Hungary

## Abstract

We aimed to estimate the proportion of the population infected with SARS-CoV-2 in the first year of the pandemic. The study population consisted of outpatient adults with mild or no COVID-19 symptoms and was divided into subpopulations with different levels of exposure. Among the subpopulation without known previous COVID-19 contacts, 4143 patients were investigated. Of the subpopulation with known COVID-19 contacts, 594 patients were investigated. IgG- and IgA-seroprevalence and RT-PCR positivity were determined in context with COVID-19 symptoms. Our results suggested no significant age-related differences between participants for IgG positivity but indicated that COVID-19 symptoms occurred most frequently in people aged between 20 and 29 years. Depending on the study population, 23.4–74.0% PCR-positive people (who were symptomless SARS-CoV-2 carriers at the time of the investigation) were identified. It was also observed that 72.7% of the patients remained seronegative for 30 days or more after their first PCR-positive results. This study hoped to contribute to the scientific understanding of the significance of asymptomatic and mild infections in the long persistence of the pandemic.

## 1. Introduction

The World Health Organization (WHO) declared the outbreak of coronavirus disease-19 (COVID-19), caused by the severe acute respiratory syndrome coronavirus-2 (SARS-CoV-2), a public health emergency of international concern under their international health regulations in January 2020 [1]. It was then declared a pandemic on 11 March 2020 [2].

The appearance of SARS-CoV-2 in Hungary, based on the initial virus detection in swab samples from two foreign university students living in Budapest, was reported in March 2020 [3]. A few days later, the viral RNA was demonstrated in small clusters of university students in Budapest, some with COVID-19 symptoms. The pandemic had reached Hungary.

Since the official recognition of SARS-CoV-2 in January 2020 [1], a significant number of studies have been published, elucidating the characteristics, diagnosis methods, possible mechanisms, and severity of the disease, as well as immune responses to SARS-CoV-2 in people suffering from COVID-19 and information on the virus itself [4,5,6,7,8,9,10,11]. However, these studies largely focused on COVID-19′s ability to cause deadly disease in humans. Much less is known about SARS-CoV-2 infections that lead to only mild COVID-19 symptoms, with no need for hospitalization or special medical treatment, or subclinical infections with no symptoms at all. However, more and more data have suggested that individuals experiencing mild disease or symptomless infections are able to spread the virus to other people, potentially causing new cases of severe COVID-19 and leading to deadly outcomes or even new waves of the pandemic [12,13,14,15].

We aimed to determine the prevalence and some characteristics [16] of asymptomatic and mild forms of COVID-19 in an outpatient subpopulation of Budapest, studied from April 2020 to March 2021. The outpatients included individuals who needed no hospitalization during the study period. The patients were evaluated for cold symptoms. Their samples were tested for antibody responses by IgG- and IgA- specific ELISA and for the presence of viral genetic material by real-time reverse-transcription polymerase chain reaction (RT-PCR).

## 2. Materials and Methods

### 2.1. Collection of Blood and Nasopharyngeal Swab Samples

The blood samples and the nasopharyngeal swab samples were collected at the Complex Medical Center (CMC) South Clinic, Budapest. CMC is a private outpatient clinic located in a residential area of the first district of Budapest, serving outpatient individuals with various health problems. The CMC has also functioned as a laboratory for SARS-CoV-2 testing in Hungary. Typically, outpatient visitors to the clinic have not included university students, and there is no university in the neighborhood. The participants of the study included (1) patients who visited the CMC clinic between April 2020 and March 2021 with mild symptoms that were potentially attributable to COVID-19; (2) patients who visited the clinic because of medical problems unrelated to COVID-19 but who were nonetheless interested in testing for possible SARS-CoV-2 infection; (3) employees of the clinic; or (4) people who needed to know their SARS-CoV-2-related status for business or travel purposes.

The collection of data was based on WHO recommendations for every sample tested in the laboratory [17]: laboratory identification number, sample collection date (dd/mm/yyyy), symptoms, type of sample, type of test, the date of the test (dd/mm/yyyy), and results (i.e., SARS-CoV-2-antibody and RT-PCR results (dd/mm/yyyy)). All the people who attended for testing signed informed consent (available in paper form), and some agreed to follow-up testing. In cases of antibody testing, a blood sample was taken. In cases of viral RNA detection, nasopharyngeal swab samples were obtained. The data collection was managed according to the current version of the Declaration of Helsinki and approved by the local Ethics Committee. All data were stored in an electronic database.

ELISA and RT-PCR were carried out at the laboratories of the Department of Microbiology and Infectious Diseases of the University of Veterinary Medicine, Budapest, and of the Veterinary Diagnostic Directorate of the National Food Chain Safety Office, Budapest. Mild COVID-19 was defined as a symptomatic disease without evidence of viral pneumonia or hypoxia [18].

### 2.2. Questionnaire

All participants were asked to complete a pseudonymized questionnaire at the study site. The questionnaire was designed according to WHO protocols [19]. It contained the name, address, and demographic data, as well as symptoms, if reported, such as fever (≥38 °C), chills, fatigue, myalgia, sore throat, cough, rhinitis, shortness of breath, chest pain, headache, anosmia, dysgeusia, and gastrointestinal symptoms. The severity of symptoms was not recorded. Yes or no questions were asked regarding coexisting diseases, such as heart disease, hypertension, diabetes, high cholesterol, asthma, allergy, or tumors. Self-reported information on SARS-CoV-2 RT-PCR tests in the past ten days and on previous close contacts with COVID-19 cases was included. Hungary’s SARS-CoV-2 vaccination program started in December 2020, but none of the participants included in the study in January–March 2021 were vaccinated.

### 2.3. SARS-CoV-2 RT-PCR

Detection of SARS-CoV-2 in nasopharyngeal wash samples was performed using RT-PCR amplification of SARS-CoV-2 N-gene fragments. Briefly, 200 µL of the nasopharyngeal washes (swabs washed in RNase-free water) were processed for RNA extraction in the Thermo Scientific™ KingFisher™ Flex Purification System (Thermo Fisher Scientific, Waltham, MA USA), using the IndiMag^®^ Pathogen Kit (QIAGEN^®^ GmbH, Hilden, Germany). Subsequently, the detection of N-gene of SARS-CoV-2 was performed using the 2019-nCoV-2 RUO kit (Integrated DNA Technologies, Inc., Coralville, IA, USA) and One-Step RT-PCR Kit (QIAGEN^®^ GmbH) on a Rotor-Gene Q real-time PCR cycler (QIAGEN^®^ GmbH). The amplification protocol consisted of a reverse transcription step at 50 °C for 30 min, a denaturation step at 95 °C for 15 min, and subsequent 45 cycles at 95 °C/56 °C/72 °C for 30/30/60 s, respectively. A positive result was defined as the amplification of N-gene in a sample, with each cycle threshold value (ct) less than 37. Virus shedding time was defined as the interval between the date of the first PCR-positive test and the date of the last PCR-positive test.

### 2.4. SARS-CoV-2 Culture, Inactivation, and Purification

Experiments with active SARS-CoV-2 were performed in the BSL-3 facilities of the Veterinary Diagnostic Directorate of the National Food Chain Safety Office, Budapest. Vero E6 cells were grown to a confluence of 80–90% and infected with the strain SARS-CoV-2/human/HUN/CMC1/2020 (GenBank OQ302121.1) at an MOI of 1 in serum-free RPMI-1640 medium, completed with non-essential amino acids (Thermo Fisher Scientific) and penicillin-streptomycin (Thermo Fisher Scientific, 10,000 U/mL). Before this, the virus titer (PFU/mL) was determined by plaque-forming assay according to the standard procedure [20]. The infected cells were incubated for four days at 37 °C with 5% CO_2_ when the cytopathic effect was visible. Virus containing supernatant was ultrafiltered using a 0.22 µm pore size filter. The filtered supernatant was inactivated with 1:2000 diluted formaldehyde solution at 25 °C for 18 h. The inactivated supernatant was purified by ultracentrifugation at 29,000 rpm (Thermo Scientific™ S58-A Fixed Angle Rotor) for 1.5 h at 4 °C. The pellet was resuspended in PBS. Inactivation was validated by inoculation of Vero E6 cell monolayers. The virus preparation was analyzed by RT-PCR assay. A Novagen^®^ BCA Protein Assay kit (MilliporeSigma, Burlington, MA, USA) was used to measure the total protein content.

### 2.5. Anti-SARS-CoV-2 IgG and IgA Antibody Testing

IgGs against SARS-CoV-2 were assessed on serum samples obtained from the participants between April and August of 2020 using a commercially-available ELISA kit (Dia.Pro Diagnostic Bioprobes S.r.l., Sesto San Giovanni, Milan, Italy), according to the manufacturer’s instructions. This assay was based on a microplate coated with a recombinant antigen of both nucleocapsid and spike proteins, and the reported sensitivity and specificity were 98% and ≥90%, respectively.

To achieve the maximal sensitivity of the antibody detection, an ELISA based on inactivated whole-virion (IWV) of SARS-CoV-2 (GenBank OQ302121.1) was developed in our laboratory. Serum samples obtained from the participants between September 2020 and March 2021 were tested using this ELISA. Coating conditions were optimized by antigen dilution and testing with convalescent sera collected from 50 SARS-CoV-2 RT-PCR-positive patients and 50 pre-pandemic control serum samples (Serum Collection of the National Public Health Center, Budapest, Hungary). The IWV-based ELISA was validated based on a comparative analysis with the Dia.Pro Diagnostic Bioprobes S.r.l. kit.

Briefly, 96-well MaxiSorp ELISA plates (Nunc, Thermo Fisher Scientific, New York, NY, USA) were coated with inactivated and purified SARS-CoV-2 whole virus antigen, diluted 1:10 in PBS overnight, then blocked with 3% bovine serum albumin (BSA) for two hours at room temperature. The plates were then washed three times with washing-diluting PBS containing 0.05% Tween 20 (Diavet Ltd., Budapest, Hungary). The serum samples, diluted 100-fold with washing-diluting buffer, were added to each well in a volume of 100 µL. After 1 h of incubation at 37 °C, the wells were washed three times. Then, 100 µL of a peroxidase-conjugated goat anti-human IgG (H + L) or goat anti-human IgA alpha chain (Abcom, Cambridge, UK), diluted 10,000-fold with PBS-Tween-20 buffer, was added to the wells, respectively. After 1 h of incubation and washing, 100 µL of tetramethylbenzidine substrate (TMB) (Diavet Ltd.) was added into the wells for 5 min. The color reaction was stopped with a 4N H_2_SO_4_ solution. The absorbance was measured at 450 nm using a FLUOstar Optima (Thermo Fisher Scientific, Waltham, MA, USA) microplate reader. Positive and negative controls were included in the respective wells in the test. Cutoff values were determined as the mean plus 2 SD of a set of 10 negative reference sera. The 100 randomly selected sera were obtained from the study participants between April and August 2020 and tested using the Dia.Pro Diagnostic Bioprobes S.r.l. ELISA kit. Samples were retested with our IWV ELISA; a 100% matching rate was observed for IgG positivity or negativity of the sera. Furthermore, 100 pre-pandemic control serum samples (Serum Collection of the National Public Health Center, Budapest, Hungary) were tested using the IWV ELISA; 8% of the sera showed a positive reaction with the IWV antigen, suggesting cross-reactivity with antibodies to other human coronaviruses, as reported by multiple studies [21].

Seroprevalence was defined as the prevalence of SARS-CoV-2-specific IgG antibodies at or above a designated OD value in the IgG ELISA. The principal analysis was based on IgG antibodies because these isotypes were elevated post-infection for a greater extended period than IgM and IgA antibodies [22]. However, since the IgA response in the early stage of the disease seemed more pronounced than IgM [23], IgA detection for the serology assessment was included.

### 2.6. Data Analysis

Data analyses were performed using Microsoft Office 365 program package. For the evaluation of the data, we used the seroprevalence estimate and 95% CIs. Categorical variables were presented as percentages and compared using the Z-probe test. A two-tailed *p*-value of less than 0.05 was statistically significant.

## 3. Results

The immunological and clinical consequences were evaluated by dividing the participants into two groups: (1) participants without known previous COVID-19 contacts and (2) participants with known previous COVID-19 contacts. Since the CMC was an outpatient clinic offering COVID-19 antibody testing, we were unable to organize a control group. Only data from the first sample collection were analyzed in both groups to limit selection bias. The characteristics of SARS-CoV-2 infection were also investigated through testing of blood and swab samples obtained, on consecutive occasions, from 33 selected participants.

### 3.1. Clinical Symptoms, Seroprevalence, and RT-PCR Positivity of Participants without Known COVID-19 Contacts

Table 1 shows the results of the first wave and the following period, with a low-level infection rate, from April to August 2020; 12.6% of the people reported symptoms, and 3.81% of the tested people had detectable levels of IgG antibodies. Table 1 also shows the participants’ results from September 2020 to March 2021, a time frame comprising, in part, the second and third waves of the pandemic in Budapest. Symptoms, serum IgG antibodies, IgA antibodies, and viral RNA were detected in 11.51%, 16.34%, 7.35%, and 8.42%, respectively. From April 2020 to March 2021, COVID-19 symptoms were observed in 11.51% of the tested participants, and SARS-CoV-2-specific serum IgG antibodies were detected in 12.94% of samples.

**Figure 1:**
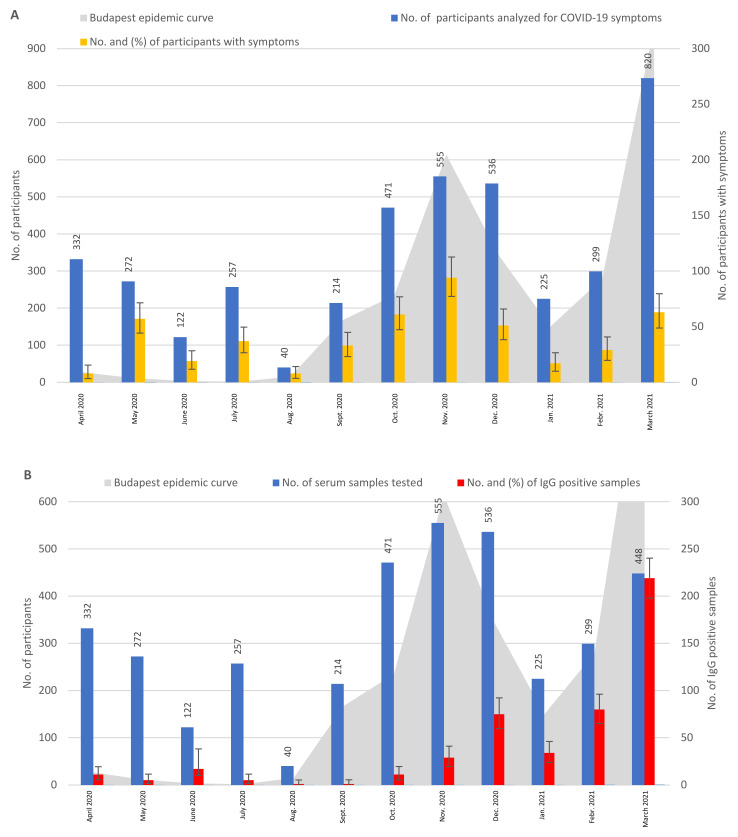

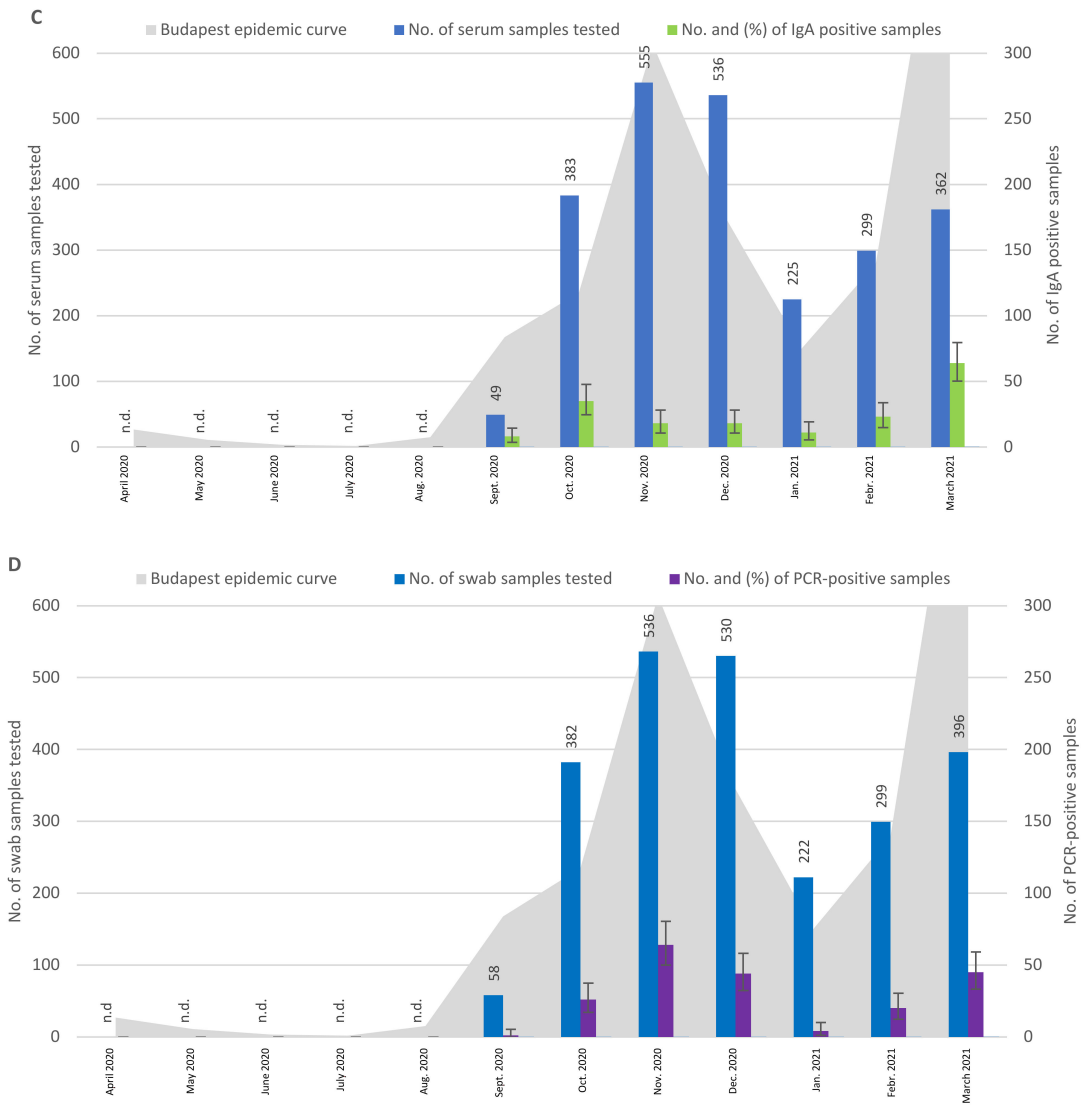
Monthly distribution and results of samples from participants with no known COVID-19 contacts. Participants are investigated for IgG and IgA antibodies using ELISA, for symptoms using a questionnaire, and for the presence of SARS-CoV-2 RNA in nasopharyngeal swab samples using RT-PCR. (**A**) Monthly distribution of participants with COVID-19 symptoms. (**B**) Monthly distribution of serum samples tested for IgG results. (**C**) Monthly distribution of serum samples tested for IgA results. (**D**) Monthly distribution of swab samples and PCR results. “n.d.”—not done.

Figure 1 shows that the monthly distribution of participants with symptoms broadly followed the epidemic curve of Budapest, especially in the second and third waves of the pandemic. The results demonstrated that participants experienced some COVID-19 symptoms, as follows: in April 2020, 2.4%; from May to August, 14.4–21.0%; from September 2020 to March 2021, 7.6–16.9% (Figure 1A).

The monthly distribution of the IgG-positive sera reflected the 2–3 week seroconversion period, with low rates in April–August 2020 (1.8–3.3%), except for a high (13.9%) IgG positivity observed in June 2020. The lowest rate of IgG positivity was observed in September (0.5%), then it slowly increased in October–November, followed by a sharp increase of 14–48.9% from December 2020 to March 2021 (Figure 1B). The monthly distribution of IgA-positive serum samples showed relatively high rates (16.3–9.1%) from September to October, then low rates from November to December (3.2–3.4%), followed by an increase in January to March 2021 (4.9–17.7%) (Figure 1C). The monthly distribution of PCR positivity showed low rates in September 2020 (1.7%) and January 2021 (1.8%) and the highest rates in November (11.9%) and March 2021 (11.4%), while relatively similar rates, between 6.7% and 8.3%, persisted in the rest of the months (Figure 1D).

The gender distribution of the IgG seroprevalence showed that, of the 2353 female participants, 296 (12.58%) were IgG-positive. Of the 1938 male participants, 263 (13.57%) were IgG-positive. These findings indicated no difference in seroprevalence between genders in the group of participants without known COVID-19 contacts.

### 3.2. Symptoms, Seroprevalence, and RT-PCR Positivity of Participants with Known COVID-19 Contacts

Table 2 shows summarized data of 182 participants tested for IgG antibodies and symptoms in April 2020, as well as 3 participants in May 2020. Of the 185 participants, 80 (43.2%) reported symptoms, and 9 patients (4.9%) were IgG-positive. From March 2020, Hungary applied physical distancing measures, such as workplace and school closures, face masks, cancellation of public events, and stay-at-home requirements, which reduced the transmission of SARS-CoV-2. Thus, no people with previous contacts asked for COVID-19 testing at CMC between May 2020 and August 2020.

However, 409 individuals previously in contact with COVID-19 cases requested COVID-19 testing between September 2020 and March 2021. These participants reported COVID-19 symptoms at a rate of 57.0%. Of the 386 tested individuals, 52 (13.5%) were shown to have SARS-CoV-2-specific IgG antibodies at or above the designated OD level to define a seropositive result. IgA antibodies were detected in 31 (9.7%) of the tested participants. Nasopharyngeal swab samples were tested by RT-PCR; 47 (14.0%) of these samples proved to be positive. During the overall study period (April 2020 to March 2021), COVID-19 symptoms were reported by 52.6% of the individuals, while 10.6% had a detectable level of IgG antibodies.

**Figure 2:**
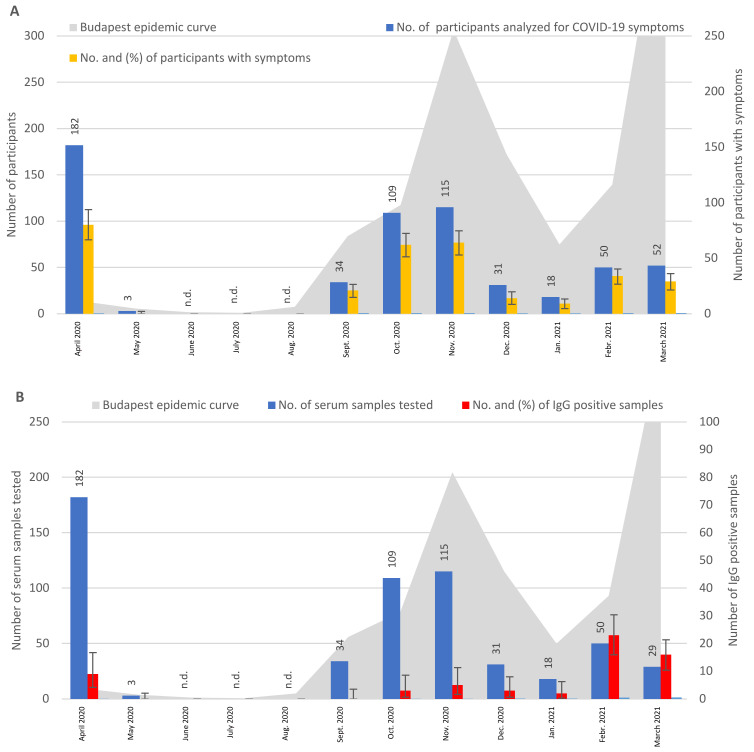

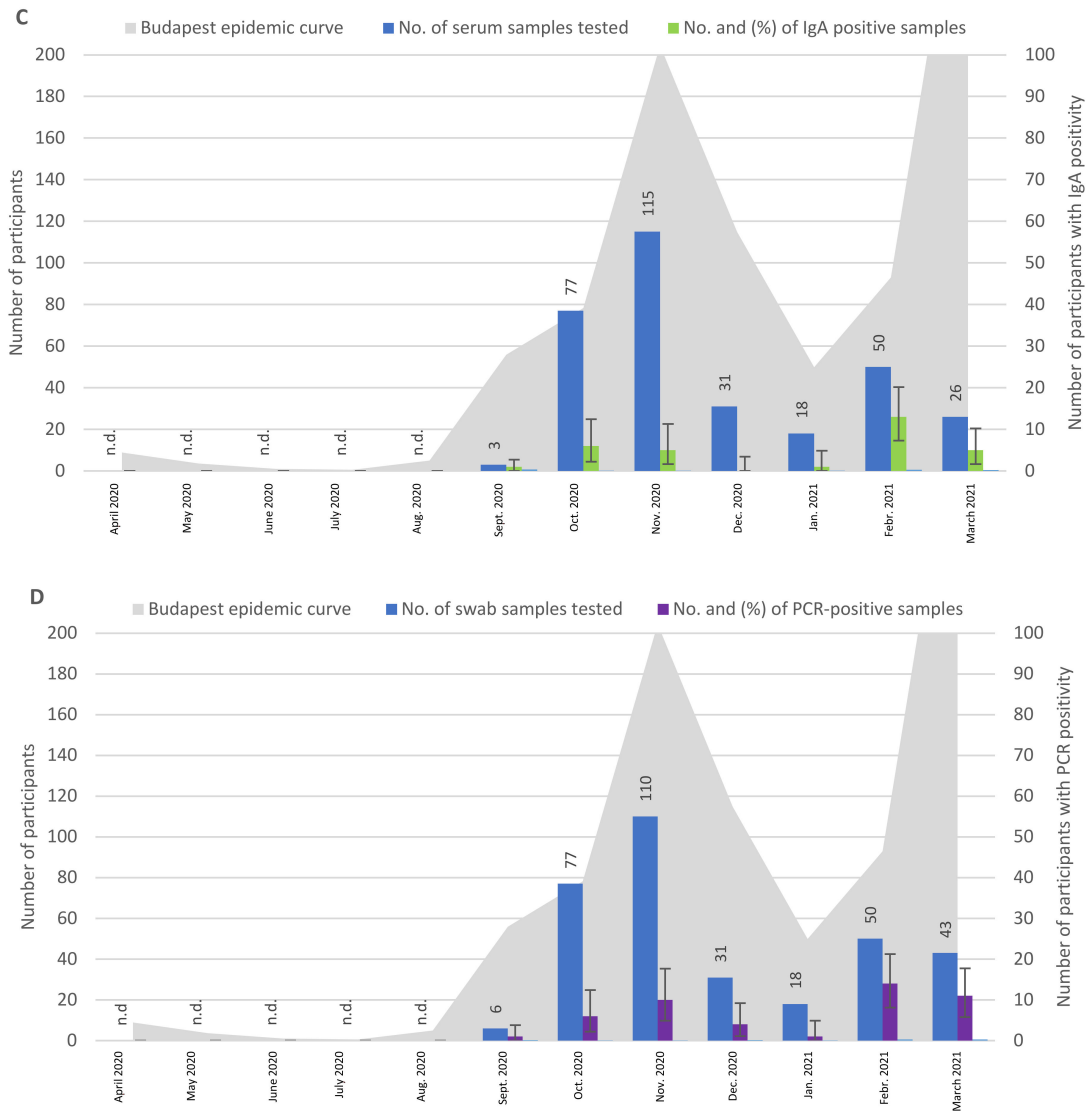
Monthly distribution and results for samples obtained from participants with known COVID-19 contacts. The participants were investigated for IgG and IgA antibodies using ELISA, for symptoms using a questionnaire, and for the presence of SARS-CoV-2 RNA in nasopharyngeal swab samples using RT-PCR. (**A**) Monthly distribution of participants with COVID-19 symptoms. (**B**) Monthly distribution of serum samples tested for IgG results. (**C**) Monthly distribution of serum samples tested for IgA results. (**D**) Monthly distribution of swab samples and PCR results. “n.d.”—not done.

As shown in Figure 2, the percentage of participants with symptoms in April 2020 was 44.0%. This varied, within a range of 45.2–68.0%, from September 2020 to March 2021 (Figure 2A). The monthly distribution of the IgG-positive sera showed 4.9% positive samples in April 2020, and the rate of seroprevalence continuously increased from September 2020 to March 2021, from 0% to 55.2% (Figure 2B). The monthly distribution of IgA-positive serum samples showed low rates (or a low number of tested people) from September 2020 to January 2021, with higher rates of positivity in February and March 2021 (26.0% and 19.2%, respectively) (Figure 2C). The monthly distribution of the PCR-positive samples varied between 16.7–5.6% from September 2020 to January 2021, with the highest rates of 28.0% and 25.6% PCR positivity detected in February and March of 2021, respectively (Figure 2D).

Similarly to participants without known COVID-19 contacts, there were no gender differences in IgG, IgA, and PCR positivity rates between male and female participants (not shown).

**Figure 3:**
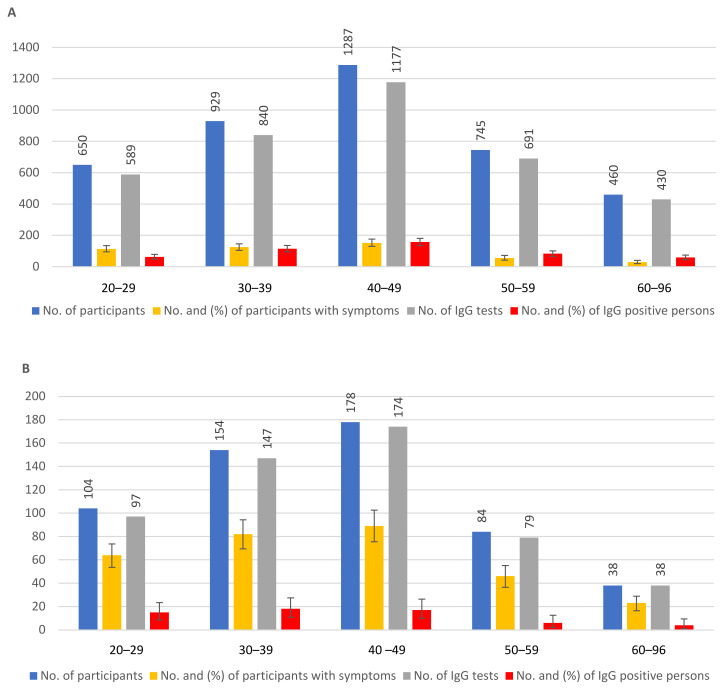
Age distribution of IgG-positive and symptom-positive people in the group of participants without known COVID-19 contacts (**A**) and in the group with known COVID-19 contacts (**B**).

The age distribution of IgG seroprevalence of participants without known COVID-19 contacts and of participants with known COVID-19 contacts (Figure 3A,B) showed that the highest number of participants who appeared for testing belonged to the 40–49 age group, followed by 30–39-year-olds, then 50–59-year-olds, 20–29-year-olds and 60–96-year-olds, in both groups. The results did not show major differences in IgG seroprevalence between these age groups for participants without COVID-19 contacts (10.7–13.7%) and with contacts (7.6–15.5%). Surprisingly, however, the highest prevalence of symptoms among groups of participants without contacts was found in the 20–29-year-old group, exceeding all older age groups (*p* < 0.05). As expected, the percentage of participants with symptoms was higher in the group with contacts than in the group without contacts: 61.5% (CI 51.5–70.9) vs. 17.5% (14.7–20.7) (20–29-year-old); 53.2% (CI 45.1–61.3) vs. 13.5% (CI 11.3–15.8), (30–39-year-old); 50.0% (42.4–57.6) vs. 11.8% (CI 10.1–13.7) (40–49-year-old); 54.8% (CI 43.5–65.7) vs. 7.5% (CI 5.7–9.7) (50–59-year-old); 60.5% (43.4–76.0) vs. 6.3% (4.3–8.9) (60–96-year-old). The *p*-value was <0.001 in all age groups (Figure 3A,B).

### 3.3. The Relation of IgG and IgA Antibodies in the Participants with or without Previous COVID-19 Contacts

In the group of participants without COVID-19 contacts, in serum samples obtained from September 2020 to March 2021, we observed a lower rate of IgA antibody responses as compared to IgG (7.35% IgA vs. 16.34% IgG) (Table 1). Similarly, in the group of participants with known COVID-19 contacts, the percentage of IgA (9.7%) was lower than that of the IgG (13.5%) responses (Table 2). The difference between IgA levels in participant groups with or without contacts was not significant (*p* = 0.138). Furthermore, IgA responses were higher at the beginning of the second wave of the pandemic in the no-contact group and higher at the beginning of the third wave in the contact group (Figure 1C and Figure 2C). This indicated that, at the beginning of the waves, more people were in an acute phase of the infection (at the time of blood sampling) than at later phases. However, the low number of IgA-positive people in both groups did not allow a statistical calculation for this observation.

### 3.4. Coexisting Diseases and COVID-19 Symptoms, in Context with PCR Positivity, in Participants with or without Previous COVID-19 Contacts

Concerning coexisting diseases such as heart disease, diabetes, cancer, asthma, and allergies, no significant differences were observed between PCR-positive and PCR-negative people in either group of participants (not shown). However, as Table 3 summarizes, in the no-contact group, PCR-positive people demonstrated a significantly higher percentage (1.5–19.6%) of all symptoms except shortness of breath, compared to PCR-negative people (0.6–1.5%). The order of the frequency of the symptoms is listed in Table 3.

Table 4 shows, that in the group of participants with known COVID-19 contacts, except for gastrointestinal manifestations, headache, and shortness of breath, COVID-19 symptoms were present in a significantly higher percentage (10.6–48.9%) in PCR-positive participants than in PCR-negative ones (3.1–35.1%). Myalgia was present in the highest percentage of PCR-positive participants, (55.3%), while headache was the most frequent symptom in PCR-negative participants (35.1%).

### 3.5. Direct Comparison of PCR Positivity and Symptoms in Participants with or without Previous COVID-19 Contacts

As shown in Table 5, 53 of 204 (26%) PCR-positive participants in the group without contacts exhibited mild COVID-19 symptoms, while 151 (74.0%) PCR-positive people were symptomless. In the group of participants with previous COVID-19 contacts, symptoms were detected in 36 of the 47 PCR-positive participants (76.6%), and 23.4% of the people in this group remained symptomless SARS-CoV-2 carriers at the time of testing. These results indicated a significantly higher percentage of participants with symptoms in the group with COVID-19 contacts.

### 3.6. Repeated Sampling and Testing of 33 Selected Participants: IgG Positivity, PCR Positivity, and Symptoms

Participants who provided samples 2–3 times after the first visit were selected. The results are summarized in Table 6, and the details are shown in Table S1.

As shown in Table 6 and Table S1, of the participants who were PCR-positive once or more than once during the 4 samples and tests, 10 (30.3%) reported symptoms (participants’ identification numbers were 1, 2, 9, 10, 13, 16, 19, 27, 29, and 33), while 23 (69.7%) participants were symptomless. Of the participants without symptoms, intermittent viral RNA shedding was detected in 3 participants (participants’ identification numbers were 7, 18, and 23), i.e., PCR positivity was followed by a PCR-negative result, which was followed by a PCR-positive result. Tests were carried out using swab samples obtained from the same person at different times during the investigation period. Shedding duration was as at least 30 days (participant’s identification number was 13). There were 9 IgG responders (27.3%), while 24 patients remained IgG-nonresponsive (72.7%), including 6 people with symptoms (participants’ identification numbers were 9, 10, 13, 16, 19, and 28).

## 4. Discussion

Epidemiological, statistical, and mathematical analyses summarized the first 1.5 years of the pandemic in Hungary. The first wave of the pandemic, concentrated in Budapest and Pest County, began in March 2020. A flat curve of COVID-19 cases was observed, ending in July. The middle of July marked the beginning of the second wave, characterized by an initial slow increase, followed by a rapidly-increasing number of cases until 19 December 2020, then slowing down again until the middle of February 2021. The third wave began on 17 February 2021 [24,25,26]. The Budapest epidemic curve showed an earlier peak of the second wave in the middle of November 2020 [27].

From April to July 2020, we tested 983 outpatients without COVID-19 contacts using ELISA (Figure 1B). An interesting finding was that, even in the first month after the official beginning of the pandemic in Hungary, i.e., in April 2020, a surprisingly high rate of IgG seroconversion (3.3%) was observed. Similarly, of the 182 sera collected in April from participants with known contacts, 4.9% were IgG-positive (Figure 2B). These results indicated that the spread of the virus in Hungary began before the presence of the virus was officially recognized in university students with typical COVID-19 symptoms, in March 2020 [3]. At the same time, we also considered the possibility of false positive reactions caused by cross-reaction with other pathogens, including other human coronaviruses [28,29]. Interestingly, SARS-CoV-2 RNA was identified in an oropharyngeal swab specimen collected from a child with suspected measles in early December 2019, 3 months before the first identified COVID-19 case in Italy [30]. In addition, the presence of SARS-CoV-2 RNA was demonstrated in the untreated wastewater of Milan, Italy, as early as mid-December 2019, suggesting the beginning of the outbreak in Europe in late autumn of 2019 [31]. Another study found antibodies against SARS-CoV-2 in human serum samples collected in the last months of 2019 (September–December) in southern Italy [32]. In our study, in the case of participants without contacts, the IgG curve in the first wave was flat, with the highest rate of IgG-positive participants (13.9%) in June. Considering the 2-week incubation time of the infection and the 2–3-week IgG seroconversion time, the 13.9% IgG positivity observed in June might have reflected the highest rate of active COVID-19 cases in May in Budapest, as reported earlier [26]. Participants with COVID-19 contacts appeared at the CMC clinic for testing from September 2020, and the highest percentages of IgG-positivity were determined from February to March 2021. IgA antibodies were determined at a lower rate than IgG antibodies, but were present in a higher percentage in the participants with known COVID-19 contacts than in participants without contacts. Additionally, higher rates of IgA antibodies were observed at the beginning of the second and third waves than at later stages (Figure 1C and Figure 2C). These results were in accordance with published results reporting 88% IgG and 10% IgA positivity rates in PCR-positive ambulatory patients [23]. A case study identified a connection between the early appearance of IgA antibodies and disease severity [23], confirming the importance of IgA detection in COVID-19 laboratory diagnosis.

In a representative, cross-sectional population survey of Hungarian individuals, the number of active infections and prevalence of seroconversion were investigated using swabs and serum samples collected in May 2020 [33]. The investigated individuals were selected from the population registry from several regions of the country. In this survey, a low active SARS-CoV-2 infection rate (0.029%) and a low overall seropositivity rate (0.68%) were identified, considering the whole country, while a higher prevalence of seropositivity (0.9%) was found in Budapest [33]. Since our study population included outpatients with mild COVID-19 symptoms and symptomless individuals visiting the CMC clinic, the 3.3–1.8% (Figure 1B) and 4.9–0% (Figure 2B) IgG seroprevalence we observed in April–May 2020 could be comparable with the 0.9% seropositivity in Budapest detected in individuals selected from the population registry by Merkely et al. [33].

Our data showed no significant differences in IgG seroprevalence between age groups, in either the contact or no-contact participation groups. Age (i.e., being older) has been accepted as a risk factor for severe COVID-19 infections. Age-dependent susceptibility to infection could explain the more common severe illnesses in the elderly, but increased disease severity could also be the result of older age and the existence of comorbidities [34,35,36]. Our results supported the second idea, i.e., that young and elderly people could be infected at a similar rate. No significant differences were found in earlier studies on susceptibility for younger adults versus older adults [34,37,38]. However, surprisingly, in our study, a higher number of individuals were found with symptoms in the 20–29-year-old age group than in older groups of no-contact participants (Figure 3A). Our interpretation of the age groups did not differentiate between people tested at the beginning or end of the year we investigated. It was reported that, early in the pandemic in the USA, COVID-19 incidence was highest among older adults, but in June–August 2020, COVID-19 incidence was highest in people aged 20 to 29 years, constituting the largest proportion of cases and indicating a decline of median age of COVID-19 cases [38]. A similar age shift was reported in Europe in 2020 [39].

We observed that, in participants with or without COVID-19 contacts who developed mild disease, the most frequent symptoms were cough, fatigue, headache, sore throat, rhinitis, and—similarly to an earlier study [13]—fever, which was 10th in the order of frequency of the 13 symptoms we documented. In a study of 656 severe cases of COVID-19, high fever (88.7%) cough (57.6%), and shortness of breath (45.6%) were the most prevalent manifestations [4]. We reported a significantly higher frequency of most of the 13 symptoms in the PCR-positive groups than in the PCR-negative groups of the investigated participants (Table 3 and Table 4).

When analyzing the 33 participants sampled and tested on consecutive occasions (Table 6 and Table S1), our serology tests determined only 4 seropositive people of the 10 outpatients with mild disease. A total of 5 people (15.1%) of the 23 were symptomless, but PCR-positive people had detectable levels of IgG antibodies 30 days after their first PCR-positive results. A much higher rate of seropositivity was reported for patients hospitalized for COVID-19; nearly 100% of these individuals exhibited IgG seropositivity [12,40,41]. One of the reasons for the low seropositivity rate in our groups of PCR-positive people might have been that virus replication in people with mild infection was limited, thus producing less antigenic stimuli for the immune system. These results indicated that there were differences between PCR-positive cases, regarding the magnitude of viral exposure and replication [42,43]. Some studies suggested that ct values would have indicated the severity of the COVID-19 cases, and thus would have been informative for the estimation of seroconversion [44]. Our results showed a significantly (*p* = 0.004) lower rate of IgG positivity in asymptomatic PCR-positive outpatients (5/23 people) than in PCR-positive ones experiencing mild symptoms of COVID-19 (4/10 people). We observed the duration of the virus shedding, for up to 30 days, in one participant with mild COVID-19 symptoms. However, since swab samples were not obtained late in our study, the virus shedding could have persisted longer than we observed. Earlier studies documented variable durations of viral shedding, from 19 days [12] or 5–16 days [45]. It should be noted, however, that PCR-positivity does not necessarily mean active virus infection; it can be the result of a previous infection with the presence of noninfectious viral RNA fragments in the swab sample.

In 3 (identification numbers 7, 18, and 23) of the 33 selected outpatients, intermittent PCR positivity was observed (Table 6 and Table S1). Similar results were reported earlier by other investigators [46,47]. False-positive RT-PCR results were highly improbable, because repeated RT-PCR assays on the same swab samples revealed consistent results. False-negative RT-PCR results could have occurred because of inadequate swab type and time since symptom or infection [46,47]. In our study, nasopharyngeal swab samples were used, which provide more reliable RT-PCR results than oropharyngeal samples. We observed that, after negative RT-PCR results, repeated testing could lead to positive PCR results, and that PCR testing at a single time point could lead to underestimation of the number of infected people without symptoms.

The monthly distribution of PCR-positive samples and symptoms showed a similar pattern in the two groups of participants, i.e., with or without COVID-19 contacts (Figure 1A,D and Figure 2A,D), indicating a relationship between the detectability of viral RNA and the development of symptoms in the patients. Direct comparison of PCR-positivity and the presence of symptoms showed that, depending on the study population, 23.4% (group with known COVID-19 contacts), 74.0% (group without known COVID-19 contacts), and 69.7% (group of consecutively sampled and tested participants) were symptomless at the time of positive PCR results (Table 5 and Table S1). However, since the symptoms were assessed at a single time point, or with a relatively short follow-up period, in some cases of consecutively tested people, it was not clear how many of these symptomless participants had presymptomatic or postsymptomatic infections, or how many were truly persistently asymptomatic. An appropriate follow-up to capture presymptomatic or postsymptomatic cases should include the maximum duration of the incubation period of 14 days, excluding presymptomatic cases, and the median duration of nasopharyngeal swab shedding of 22 days [48] or 30 days (our observation) to exclude postsymptomatic cases [49]. Nevertheless, identifying and managing asymptomatic individuals and their close contacts to control possible outbreaks would be of great significance to the public health [50]. In an earlier study, the proportion of patients with asymptomatic infections was 20.8% [12].

Limitations of our study included that the serum and swab samples were not always obtained according to a well-designed plan, but were collected according to the availability and request of the participants. Individual differences in time in serum and swab sampling since symptom onset could have influenced the RT-PCR and ELISA results in mild COVID-19 cases. This could be true for asymptomatic people, as well. Some imperfections arose from the limited availability of certain laboratory diagnostic methods at the very early phase of the pandemic, i.e., IgA-specific ELISA and RT-PCR were not available at the beginning of the pandemic, or at the beginning of our study, but were later included. Furthermore, the number of participants in certain settings was low; thus, calculations of percentages might not be accurate, and the results should be confirmed.

More work needs to be done to estimate the rate of asymptomatic and mild infections with SARS-CoV-2 in various clinical settings and the general population. It is of great importance to understand the significance of these infections in the persistence, duration, and repeated outbreaks of the pandemic, as well as their possible roles in achieving herd immunity in the population. These goals are challenging for many reasons, including the emerging mutated variants of the virus, which drive new infection waves. In this regard, our data could provide a basis for comparing the experiences of different epidemic waves, with particular reference to the seroprevalence of symptomless SARS-CoV-2 carriers.

## 5. Conclusions

Our observational study on symptomless or mildly infected people began at a very early stage of the pandemic. We observed a 3.3–4.9% seroprevalence in April 2020, indicating a fast spread, or early appearance, of SARS-CoV-2 in Budapest. There was a significantly higher IgG seroprevalence in the group of participants without COVID-19 contacts in the one-year period from April 2020 to March 2021 in Budapest, compared with registered COVID-19 cases in Budapest during the same time [27]. Our results suggested no significant age-related differences between participants for IgG positivity, but indicated that COVID-19 symptoms were most frequent in people aged between 20 and 29. Depending on the study population, a high proportion of PCR-positive people, who were symptomless SARS-CoV-2 carriers at the time of the investigation, were identified. It was also noted that these patients remained seronegative within 30 days (or more) after their first PCR-positive results.

## Figures and Tables

**Table 1:** Symptoms, seroprevalence, and RT-PCR positivity of participants without known COVID-19 contacts.

Characteristics	Total No. of Tested People	Time Period	No. of People with Positive RT-PCR-Results	Percentage of Positive People (95% CI)
Symptoms	1023 of 1023	April 2020–August 2020	129	12.6 (10.64–14.80)
IgG antibodies	1023 of 1023	April 2020–August 2020	39	3.8 (2.72–5.17)
Symptoms	3120 of 3120	September 2020–March 2021	348	11.1 (10.07–12.31)
IgG antibodies	2748 of 3120	September 2020–March 2021	449	16.3 (14.98–17.78)
IgA antibodies	2409 of 3120	September 2020–March 2021	177	7.3 (6.34–8.46)
Viral RNA	2423 of 3120	September 2020–March 2021	204	8.4 (7.34–9.6)
Symptoms	4143 of 4143	April 2020–March 2021	477	11.5 (10.56–12.52)
IgG antibodies	3771 of 4143	April 2020–March 2021	488	12.9 (11.89–14.05)

**Table 2:** Symptoms, seroprevalence, and RT-PCR positivity of participants with known COVID-19 contacts.

Characteristics	Total No. of Tested People	Time Period	No. of People with Positive RT-PCR-Results	Percentage of Positive People (95% CI)
Symptoms	185 of 185	April 2020–May 2020	80	43.2 (35.99–50.71)
IgG antibodies	185 of 185	April 2020–May 2020	9	4.9 (2.25–9.03)
Symptoms	409 of 409	September 2020–March 2021	233	57.0 (52.01–61.82)
IgG antibodies	386 of 409	September 2020–March 2021	52	13.5 (10.23–17.29
IgA antibodies	320 of 409	September 2020–March 2021	31	9.7 (6.68–13.47)
Viral RNA	335 of 409	September 2020–March 2021	47	14.0 (10.46–18.16)
Symptoms	594 of 594	April 2020–March 2021	313	52.6 (48.59–56.77)
IgG antibodies	571 of 594	April 2020–March 2021	61	10.6 (8.27–13.51)

**Table 3:** Clinical characteristics of 2423 PCR-tested participants without COVID-19 contacts.

Symptoms *	PCR-Positive Participants	%	PCR-Negative Participants	%	*p*-Value
	*n* = 204		*n* = 2219		
cough	40	19.6	68	3.1	<0.001
fatigue	33	16.2	98	4.4	<0.001
headache	33	16.2	125	5.6	<0.001
sore throat	27	13.2	80	3.6	<0.001
rhinitis	24	11.8	84	3.8	<0.001
chills	21	10.3	32	1.4	<0.001
myalgia	23	11.3	38	1.7	<0.001
anosmia	15	7.4	19	0.9	<0.001
dysgeusia	15	7.4	19	0.9	<0.001
fever	14	6.9	19	0.9	<0.001
chest pain	7	3.4	22	1.0	0.002
gastrointestinal symptoms	6	2.9	13	0.6	<0.001
shortness of breath	3	1.5	13	0.6	0.135

* The number and % of the participants with the indicated symptoms are shown.

**Table 4:** Clinical characteristics of PCR-tested participants with known COVID-19 contacts.

Symptoms *	PCR-Positive Participants		PCR-Negative Participants		
	*n* = 47	%	*n* = 288	%	*p*-Value
sore throat	22	46.8	59	20.5	<0.001
cough	20	42.6	41	14.2	<0.001
fatigue	23	48.9	57	19.8	<0.001
rhinitis	20	42.6	57	19.8	0.001
headache	20	42.6	101	35.1	0.322
myalgia	26	55.3	22	7.6	<0.001
anosmia	11	23.4	9	3.1	<0.001
dysgeusia	11	23.4	9	3.1	<0.001
chills	12	25.5	20	6.9	<0.001
fever	10	21.3	9	3.1	<0.001
chest pain	5	10.6	8	2.8	0.010
shortness of breath	5	10.6	15	5.2	0.145
gastrointestinal symptoms	0	0.0	19	6.6	0.070

* The number and % of the participants with the indicated symptoms are shown.

**Table 5:** Direct comparison of PCR positivity with COVID-19 symptoms in groups of participants with or without COVID-19 contacts from September 2020 to March 2021.

Groups	No. of PCR-Positive People	No. and Percentage PCR-Positive People with Symptoms	No. and Percentage of PCR-Positive People without Symptoms
Participants without COVID-19 contact	204	53 (26.0) *^,+^	151 (74.0) ^+^
Participants with COVID-19 contact	47	36 (76.6) *^,×^	11 (23.4) ^×^

* *p* < 0.001, ^+^ *p* < 0.001, ^×^ *p* < 0.001; * The PCR-positive people were divided according to the presence or absence of symptoms, as recorded in the questionnaire.

**Table 6:** Results of samples obtained from participants, sampled and tested 3–4 times.

Participants	PCR-Positive	IgG-Positive	IgG-Negative
10 with symptoms	4 (1 of 3–4 samples was positive)	2	2
	6 (more than 1 of 3–4 samples were positive)	2	4
23 without symptoms	8 (1 of 3–4 samples was positive)	3	5
	12 (more than 1 of 3–4 samples were positive)	1	11
	3 (intermittent positive and negative samples)	1	2

Samples obtained repeatedly from participants and tested using PCR and ELISA. A significantly lower (*p* = 0.004) rate of IgG positivity is observed in participants without symptoms (5/23) compared to participants with symptoms (4/10).

## Data Availability

Not applicable.

## Supplementary Materials

The following supporting information can be downloaded at: https://www.mdpi.com/article/10.3390/tropicalmed8040204/s1, Table S1: Results of samples obtained at the first visit of the participants at the CMC Clinic and days (in brackets) after the first visit.
