# Supplementary material for: Asymptomatic and Mild SARS-CoV-2 Infections in a Hungarian Outpatient Cohort in the First Year of the COVID-19 Pandemic
Source: Trop Med Infect Dis. 2023 Mar 29;8(4):204. doi: 10.3390/tropicalmed8040204 (PMC10146718; doi:10.3390/tropicalmed8040204)
Supplement: Supplementary file 1 [file tropicalmed-08-00204-s001.zip › tropicalmed-2150573-supplementary.pdf]

Table S1. Results of the samples obtained at the first visit and days (in brackets) after the first visit.

Participant identification number	Age (Years)	Sex	Test	Date of first visit	Visit 1	2	3	4
**1***	37	F	PCR		+	-	-	nd
				10.15.20		(53d)	(63d)	
			IgG		-	+(0.695)	nd	nd
**2***	25	F	PCR		+	-	-	nd
				10.28.20		(12d)	(102d)	
			IgG		-	-	+(1.093)	
**3**	42	F	PCR		+	+	-	nd
				10.30.20		(6d)	(35d)	(129d)
			IgG		-	-	-	-
**4**	44	M	PCR		+	-	-	nd
				11.02.20		(18d)	(21d)	
			IgG		-	-	-	nd
**5**	51	M	PCR		+	+	+	-
				11.02.20		(4d)	(17d)	(80d)
			IgG		-	-	-	-
**6**	57	M	PCR		+	+	-	nd
				11.06.20		(6 d)	(42d)	
			IgG		-	-	-	nd
**7**	32	F	PCR		+	-	+	nd
				11.11.20		(60d)	(105d)	
			IgG		-	-	-	nd
**8**	26	M	PCR		+	+	+	-
				11.11.20		(5d)	(14d)	(62d)
			IgG		-	-	-	-
**9***	53	M	PCR		+	-	-	nd
				11.11.20		(15d)	(30d)	
			IgG		-	-	-	nd
**10***	53	F	PCR		+	+	-	-
				11.13.20		(17)	(24)	(27)
			IgG		-	-	-	-
**11**	45	F	PCR		+	-	-	nd
				11.18.20		(12d)	(30d)	
			IgG		-	-	+(0.608)	nd
**12**	27	F	PCR		-	-	+	nd
				11.18.20		(8d)	(47d)	
			IgG		-	-	-	nd
**13***	33	F	PCR	11.18.20	+	+	+	nd

						(15d)	(30d)	
			IgG		-	-	-	nd

Participant identification number	Age (Years)	Sex	Test	Date of first visit	Visit 1	2	3	4
**14**	27	M	PCR		-	+	+	-
				11.19.20		(12d)	(25d)	(32d)
			IgG		-	-	-	-
**15**	34	F	PCR		+	-	-	nd
				11.20.20		(6d)	(79d)	
			IgG		-	-	-	nd
**16***	49	F	PCR		+	+	+	-
				11.24.20		(14d)	(21d)	(26d)
			IgG		-	-	-	-
**17**	32	M	PCR		-	+	+	nd
				11.25.20		(7d)	(27d)	
			IgG		-	-	-	nd
**18**	42	F	PCR		+	-	+	
				11.26.20		(12d)	(15d)	nd
			IgG		-	-	-	nd
**19***	45	M	PCR		+	nd	+	nd
				11.30.20		(10d)	(24d)	
			IgG		-	-	-	nd
**20**	20	M	PCR		+	+	-	nd
				12.01.20		(14d)	(21d)	
			IgG		-	-	-	nd
**21**	27	M	PCR		+	+	+	nd
				12.01.20		(9d)	(14d)	
			IgG		-	-	-	nd
**22**	32	M	PCR		+	+	-	nd
				12.03.20		(12d)	(19d)	
			IgG		-	-	-	nd
**23**	43	F	PCR		+	-	+	-
				12.04.20		(14d)	(19d)	(25d)
			IgG		-	-	-	+ (0.339)
**24**	43	M	PCR		+	+	-	nd
				12.04.20		(10d)	(18d)	
			IgG		-	-	-	nd
**25**	34	M	PCR		+	+	-	nd
				12.07.20		(14d)	(21d)	
			IgG		-	-	-	nd
**26**	27	F	PCR		-	+	-	nd
				12.21.20		(16d)	(46d)	
			IgG		+(0.415)	-	+(0.91)	nd
**27***	55	M	PCR	01.25.21	-	-	-	+

Participant Identification number	Age (Years)	Sex	Test	Date of first visit	Visit 1	Visit 2	Visit 3	Visit 4
						(2d)	(24d)	(27d)
			IgG		-	-	-	-
**28**	58	F	PCR		-	-	+	nd
				01.25.21		(2d)	(23d)	
			IgG		-	-	-	nd
**39***	46	M	PCR		-	-	+	+
				01.27.21		(6d)	(19d)	(30d)
			IgG		-	-	-	+(0.817)
**30**	52	F	PCR		+	+	+	nd
				02.11.21		(4d)	(14d)	
			IgG		-	-	-	nd
**31**	68	F	PCR		+	-	-	nd
				02.15.21		(10d)	(17d)	
			IgG		-	+(1.348)	-	nd
**32**	50	F	PCR		-	-	+	nd
				02.15.21		(11d)	(15d)	
			IgG		-	-	-	nd
**33***	45	F	PCR		+	+	-	-
				02.16.21		(3d)	(10d)	(15d)
			IgG		-	-	-	+(1.640)

* Participants with symptoms
nd=not done
